# A case of renal cortical necrosis likely caused by disseminated intravascular coagulation after acute type A aortic dissection repair

**DOI:** 10.1093/jscr/rjaf793

**Published:** 2025-10-09

**Authors:** Kotaro Mukasa, Yasunori Yakita, Ryosuke Marushima, Shinichiro Abe, Soichi Asano

**Affiliations:** Department of Cardiovascular Surgery, Chiba Cardiovascular Center, Ichihara 290-0512, Japan; Department of Cardiovascular Surgery, Chiba Cardiovascular Center, Ichihara 290-0512, Japan; Department of Cardiovascular Surgery, Chiba Cardiovascular Center, Ichihara 290-0512, Japan; Department of Cardiovascular Surgery, Chiba Cardiovascular Center, Ichihara 290-0512, Japan; Department of Cardiovascular Surgery, Chiba Cardiovascular Center, Ichihara 290-0512, Japan

**Keywords:** aortic dissection, disseminated intravascular coagulation, renal cortical necrosis

## Abstract

Acute type A aortic dissection carries a risk of disseminated intravascular coagulation (DIC) due to blood exposure in the false lumen, surgical factors, and false lumen thrombosis. We report the case of a 70-year-old woman who underwent total arch replacement with frozen elephant trunk implantation. Postoperatively, she developed DIC complicated by acute kidney injury and cerebral infarction. Contrast-enhanced computed tomography revealed the reverse rim sign, indicating renal cortical necrosis. Ultimately, irreversible renal failure necessitated maintenance hemodialysis. DIC associated with aortic dissection can lead to multi-organ failure from systemic microthrombosis and requires vigilant monitoring.

## Introduction

Acute type A aortic dissection (ATAAD) is a life-threatening condition characterized by complications such as rupture and organ malperfusion, necessitating prompt surgical intervention. Turbulent blood flow within the false lumen exposed during dissection is known to activate the coagulation cascade, thereby increasing the risk of disseminated intravascular coagulation (DIC). Additionally, extracorporeal circulation and hypothermia associated with surgery may further promote DIC. DIC can result in irreversible damage to multiple organs through systemic microthrombotic embolism. We herein report a case of renal cortical necrosis (RCN)-associated acute kidney injury following emergent total arch replacement and frozen elephant trunk (FET) implantation for Stanford type A aortic dissection. The diagnosis of RCN was established based on characteristic findings on contrast-enhanced computed tomography (CT), in the context of postoperatively developed DIC.

**Figure 1:**
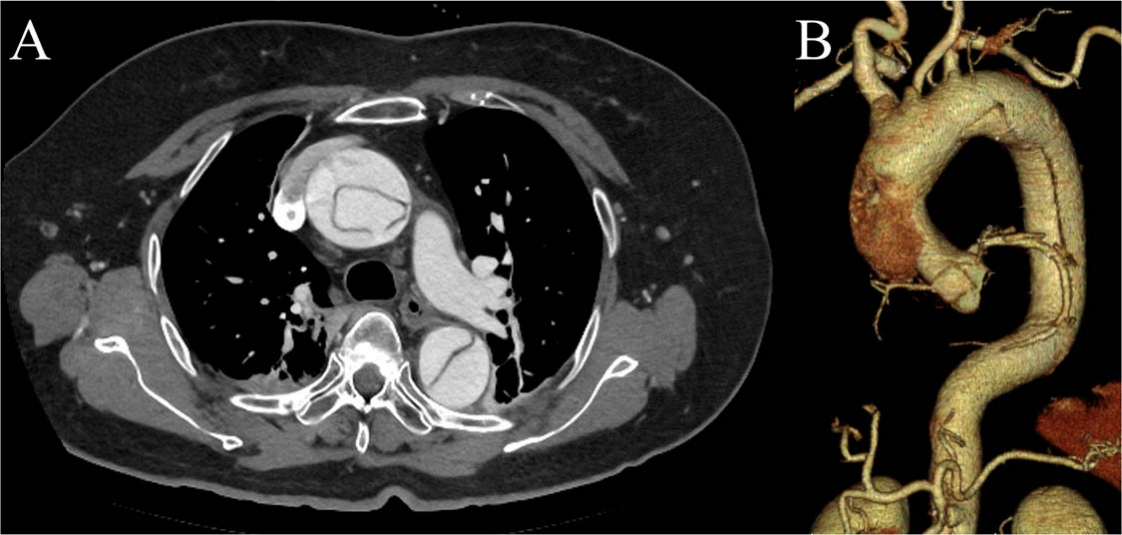
Preoperative contrast-enhanced CT images. (A) Stanford type A acute aortic dissection with the primary entry tear extending from the ascending aorta to the aortic arch, showing a patent false lumen. (B) Three-dimensional reconstructed image.

**Figure 2:**
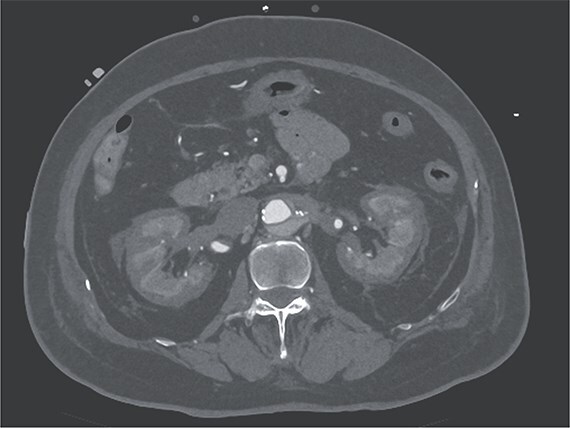
Contrast-enhanced CT image acquired on postoperative Day 2, showing the reverse rim sign.

## Case report

**Figure 3:**
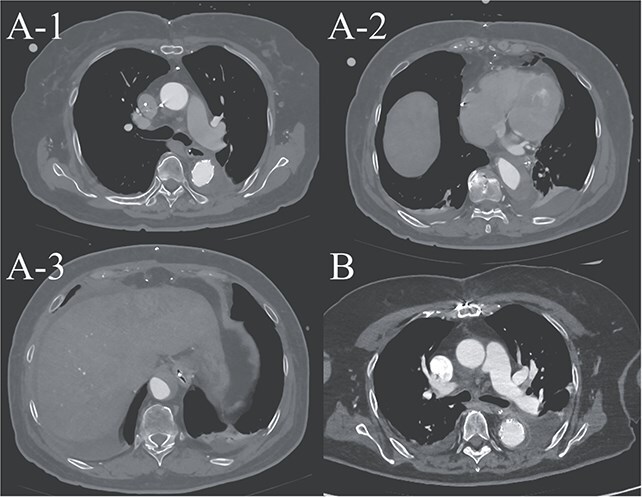
(A-1–3) contrast-enhanced CT image acquired on postoperative Day 2, showing advanced false lumen thrombosis. (B) Contrast-enhanced CT image acquired on postoperative Day 21, demonstrating further remodeling of the false lumen. This image is at approximately the same level as A-1, and the false lumen's thickness is clearly reduced.

**Figure 4:**
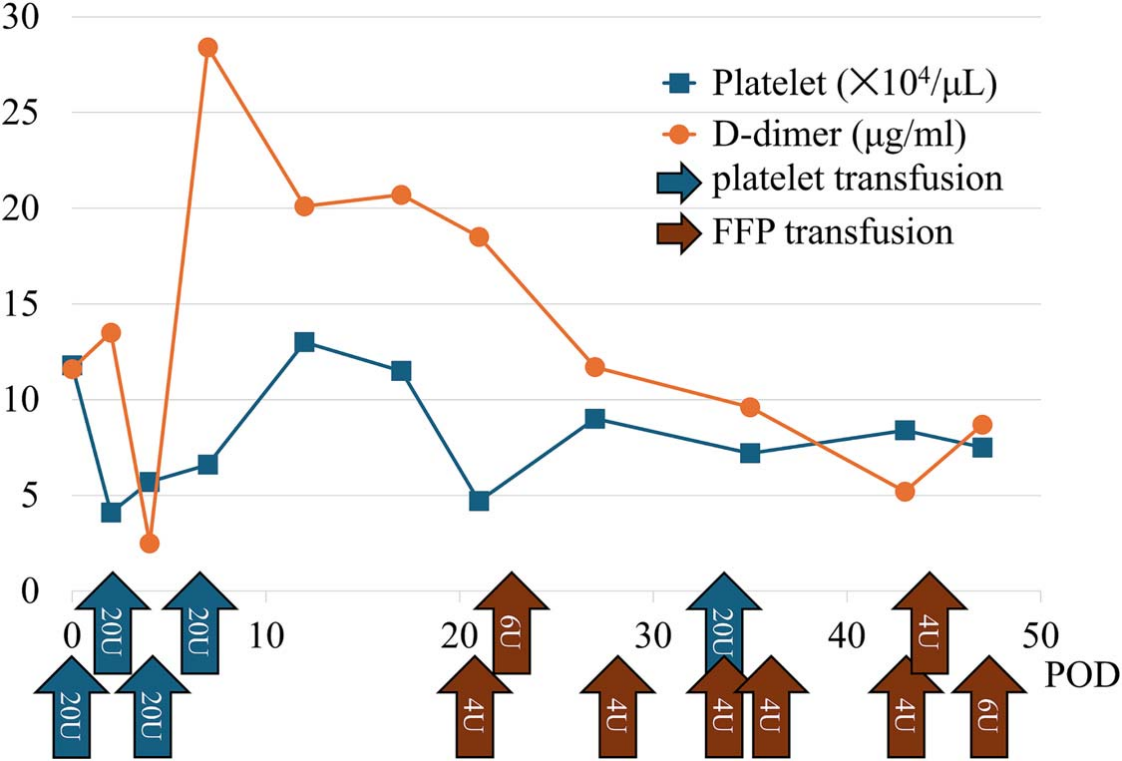
Graph showing the trends in postoperative coagulation parameters and the transfusion volumes of fresh frozen plasma and platelets. This indicates a prolonged consumption of coagulation factors postoperatively.

**Figure 5:**
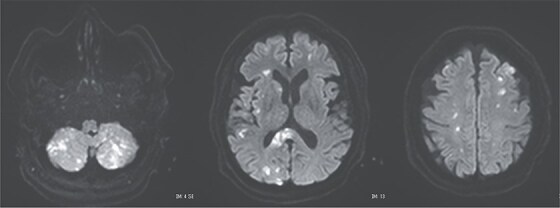
Postoperative magnetic resonance imaging showing diffuse cerebral infarctions.

A 70-year-old woman presented to the emergency department with sudden-onset chest and back pain. Contrast-enhanced CT revealed an ATAAD extending from the ascending aorta to the bilateral common iliac arteries. The primary entry tear extended from the ascending aorta into the aortic arch, and the false lumen remained patent throughout its entire peripheral extent (Fig. 1). Both renal arteries originated from the true lumen, and there was no malperfusion. Vital signs were stable, and the patient was not in shock. The patient underwent emergency total arch replacement with FET implantation using a 27 × 90 mm FROZENIX four-branched graft (Japan Lifeline Co., Ltd, Tokyo, Japan). The procedure was completed in 7 h and 6 min without intraoperative complications. Postoperatively, urine output declined markedly. Serum creatinine increased from a baseline of 0.81 to 2.61 mg/dl on postoperative Day 1. Diuretics failed to improve urine output, and renal replacement therapy was initiated. Contrast-enhanced CT on postoperative Day 2 showed no signs of renal malperfusion. Although the renal medulla exhibited normal enhancement, a diffuse non-enhancing area throughout the renal cortex—referred to as the “reverse rim sign”—was observed, consistent with RCN (Fig. 2). Thrombosis of the false lumen in the descending thoracic and thoracoabdominal aorta had progressed (Fig. 3). The patient developed persistent coagulopathy, with sustained hypofibrinogenemia and thrombocytopenia. On postoperative Day 2, the platelet count was 4.1 × 10^4^/μl, D-dimer 13.5 μg/ml, and PT-INR 1.54, yielding an International Society on Thrombosis and Hemostasis DIC score of 6, consistent with overt DIC. Anticoagulation therapy with heparin and warfarin was administered. Ultimately, by postoperative Day 50, a total of 36 units of fresh frozen plasma and 100 units of platelets had been transfused; however, coagulation parameters gradually normalized thereafter, and no further transfusions were required (Fig. 4). A follow-up CT on postoperative Day 21 demonstrated further remodeling of the thrombosed false lumen. Brain magnetic resonance imaging performed on postoperative Day 9 to investigate delayed emergence revealed multiple scattered small acute ischemic infarctions involving the cerebellum, brainstem, and cerebral cortex (Fig. 5). These were not attributed to large-vessel occlusion but rather to systemic microthrombotic emboli. Despite supportive care, renal function did not recover, and maintenance dialysis was initiated. The patient was transferred to another hospital on postoperative Day 182.

## Discussion

This case illustrates the development of DIC following total arch replacement with FET implantation for ATAAD, leading to systemic microthrombotic embolism with resultant RCN and diffuse cerebral infarctions. In ATAAD, exposure of circulating blood to the dissected medial layer promotes coagulation cascade activation [1]. Postoperative factors such as cardiopulmonary bypass, heparin administration, and hypothermia further exacerbate the coagulopathic state. In particular, FET implantation is known to facilitate early remodeling of the distal aorta, and the associated extensive thrombosis may accelerate the consumption of coagulation factors, precipitating DIC [2]. Postoperative DIC is associated with increased mortality and serves as an indicator of poor prognosis [3].

DIC is frequently associated with acute kidney injury. Among them, RCN represents the most severe form, frequently resulting in irreversible renal failure. RCN is characterized by widespread coagulative necrosis due to decreased cortical perfusion. The renal cortex is more vulnerable to ischemia than the medulla because of its limited collateral circulation [4]. The “reverse rim sign,” defined by preserved medullary enhancement with cortical non-enhancement on contrast-enhanced CT, is a hallmark and highly specific imaging finding for RCN [5]. In DIC-associated RCN, systemic microthrombosis and regional hypoperfusion often cause irreversible cortical necrosis, leading to the need for long-term dialysis or kidney transplantation. In this case, RCN developed despite the absence of prolonged hypotension or renal artery malperfusion, suggesting a strong association with DIC. Diffuse RCN, as seen in the present case, is associated with worse renal outcomes than patchy RCN [6]. The advantage of diagnosing RCN, as opposed to typical acute kidney injury, is the ability to predict poor renal prognosis early on, allowing for earlier preparation for maintenance dialysis.

Postoperative DIC following ATAAD repair can compromise major organ function, including the kidneys and brain. Prompt diagnosis and appropriate anticoagulation may be essential to improve patient outcomes. Although recombinant thrombomodulin has been reported to be effective in DIC, its use may be contraindicated in cases with high bleeding risk [7]. In our case, the presence of widespread cerebral infarctions raised concern for hemorrhagic transformation; thus, thrombomodulin was not administered.

## Data Availability

Not applicable.
